# Biphasic Clinical Course Revealing Lemierre's Syndrome Following Acute Pharyngotonsillitis: A Case Report

**DOI:** 10.7759/cureus.110268

**Published:** 2026-06-04

**Authors:** Takayuki Yoshioka, Rika Satou

**Affiliations:** 1 General Internal Medicine, Kobe Rousai Hospital, Kobe, JPN

**Keywords:** cavitary nodule, fusobacterium necrophorum, internal jugular vein thrombosis, lemierre's syndrome, pharyngotonsillitis, septic emboli

## Abstract

Lemierre's syndrome is a life-threatening septic thrombophlebitis that typically follows an oropharyngeal infection and is most commonly caused by *Fusobacterium necrophorum (F. necrophorum)*. We report a case of a previously healthy woman in her 20s who developed septic shock and acute kidney injury after a recurrent episode of acute pharyngotonsillitis. She was initially treated at a local clinic with a short course of oral third-generation cephalosporin for presumed group A streptococcal pharyngotonsillitis, resulting in temporary clinical improvement. However, she subsequently experienced a recurrence of fever and sore throat, necessitating emergency hospitalization. She was treated immediately with intensive intravenous ampicillin/sulbactam and vasopressor support. A contrast-enhanced CT several days later demonstrated septic pulmonary emboli, and blood cultures subsequently yielded *F. necrophorum*. Although internal jugular vein (IJV) thrombosis was not evident, a diagnosis of Lemierre's syndrome was established based on antecedent pharyngotonsillitis, *F. necrophorum* bacteremia, and metastatic septic emboli. This report suggests that short-course oral cephalosporin therapy for presumed group A streptococcal pharyngotonsillitis does not necessarily prevent the subsequent development of invasive* F. necrophorum* infection. It may also delay the recognition of Lemierre's syndrome.

## Introduction

Lemierre’s syndrome is a rare but potentially life-threatening complication of acute oropharyngeal infection that predominantly affects otherwise healthy adolescents and young adults [1-3]. It typically develops when an antecedent viral or bacterial pharyngitis disrupts the pharyngeal mucosal barrier, allowing pathogens to invade the lateral pharyngeal space and subsequently cause septic thrombophlebitis with the formation of septic emboli [4,5]. The definition of Lemierre’s syndrome has varied across the literature, reflecting differences in the clinical features used for diagnosis. It is generally characterized by a preceding oropharyngeal infection, internal jugular vein (IJV) thrombosis, anaerobic bacteremia most commonly caused by *Fusobacterium necrophorum (F. necrophorum)*, and septic emboli, typically involving the lungs [1,2,4,6-8]. Although IJV thrombosis has traditionally been regarded as a hallmark of the syndrome, it is not required for the diagnosis when characteristic clinical features are present [1,2,7,8].

Despite substantial advances in antimicrobial therapy, studies published over the past two decades have reported mortality rates of approximately 2-9% [1,2,6,9]. Beyond age, few risk factors have been consistently identified. Possible predisposing factors include recurrent tonsillitis leading to tonsillar scarring, Epstein-Barr virus infection, and other proposed host or microbial factors; however, their exact roles remain unclear [1,4]. Atypical presentations of Lemierre’s syndrome have also been reported, including abdominal variants associated with portal vein thrombosis, cases caused by pathogens such as *Klebsiella pneumoniae* and methicillin-resistant* Staphylococcus aureus *(MRSA), and cases arising from infectious sites other than the oropharynx [10-13].

In Lemierre's syndrome, initial symptoms such as sore throat and fever may temporarily improve before progression to systemic illness [1,4,8]. Although corticosteroid therapy administered for presumed viral pharyngitis has been reported to potentially exacerbate progression to Lemierre's syndrome in some cases, clinical deterioration after apparent improvement with antimicrobial therapy remains relatively uncommon [2,6,14]. This biphasic clinical course may therefore be particularly challenging to recognize in routine clinical practice. We report a case of Lemierre’s syndrome that became clinically apparent after a transient improvement of acute pharyngotonsillitis, underscoring the diagnostic challenges posed by a biphasic clinical course.

## Case presentation

A previously healthy woman in her 20s developed fever and a sore throat approximately two weeks before presentation. She was diagnosed with acute streptococcal pharyngotonsillitis at a local clinic and was treated with a five-day course of cefditoren pivoxil (CDTR-PI). Although her symptoms initially improved, fever and sore throat recurred four days following the completion of the five-day course of CDTR-PI, accompanied by right-sided neck pain. Her condition progressively worsened, leading to severe dysphagia, and she was subsequently transported to our hospital by ambulance. The clinical course is summarized in Figure 1.

**Figure 1:**
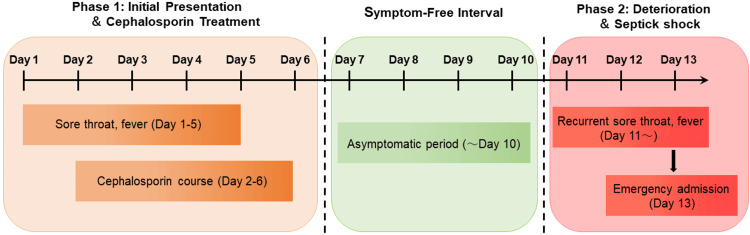
Timeline of the biphasic clinical course The patient initially presented with acute pharyngotonsillitis and received a five-day course of cephalosporin, which led to temporary clinical improvement. This was followed by a four-day asymptomatic period. Subsequently, the patient developed recurrent fever and rapidly progressed to septic shock. This figure was created using Microsoft PowerPoint (Microsoft, Redmond, WA)

On arrival, the patient was alert but appeared severely ill. Her temperature was 39.9 ℃, blood pressure 80/40 mmHg, heart rate 129 beats per minute, and oxygen saturation 96% on room air. Physical examination revealed bilateral tonsillar swelling with white exudates, anterior cervical lymphadenopathy, and no cough. The modified Centor (McIsaac) score was 4. Cardiopulmonary examination was unremarkable, and there were no signs of meningismus, skin rash, or joint involvement. Laboratory findings demonstrated markedly elevated inflammatory markers, impaired renal function, and coagulation abnormalities suggestive of evolving disseminated intravascular coagulation (Table 1).

**Table 1:** Initial laboratory data on admission The laboratory findings demonstrated elevated inflammatory markers and abnormalities suggestive of acute kidney injury and disseminated intravascular coagulation (DIC). Values outside the normal range are shown in bold

Parameter	Result	Normal range	Units
White blood cells	16,650	3,100-8,400	μL
Hemoglobin	11.9	12.1-14.5	g/dL
Platelet	156,000	145,000-329,000	μL
Serum creatinine	2.03	0.46-0.79	mg/dL
Blood urea nitrogen	38.2	8.0-21.0	mg/dL
C-reactive protein	22.49	< 0.3	mg/dL
Procalcitonin	41.88	< 0.05	ng/mL
Prothrombin time	14.2	10.0-13.5	sec
Activated partial thromboplastin time	36	24-34	sec
D-Dimer	8.9	< 1.0	μg/mL
Fibrin degradation products	17.8	< 5	μg/mL

A rapid antigen detection test (RADT) for group A *Streptococcus* performed on a throat swab at presentation (hospital day one), during the second phase of illness following a transient clinical improvement, was positive. Acute streptococcal pharyngotonsillitis was therefore suspected, although the possibility of mixed infection or involvement of anaerobic pathogens was also considered. During hospitalization, platelet counts further decreased, and coagulation abnormalities progressed, fulfilling the diagnostic criteria for acute-phase disseminated intravascular coagulation on hospital day two. Intravenous ampicillin/sulbactam (ABPC/SBT) was initiated upon admission, along with vasopressor support using norepinephrine.

Although inflammatory markers gradually improved and norepinephrine was discontinued on hospital day three, a persistent high-grade fever of more than 38 ℃ was observed. A contrast-enhanced CT performed on hospital day four revealed a cavitary nodule in the left upper lobe, consistent with septic pulmonary emboli (Figure 2). Subsequently, blood cultures processed using standard microbiological methods at our institution yielded *F. necrophorum*, which was identified by matrix-assisted laser desorption/ionization time-of-flight (MALDI-TOF) mass spectrometry. Based on the presence of *F. necrophorum* bacteremia and septic pulmonary emboli following pharyngotonsillitis, a diagnosis of Lemierre's syndrome was established despite the lack of evidence of IJV thrombosis on imaging.

**Figure 2:**
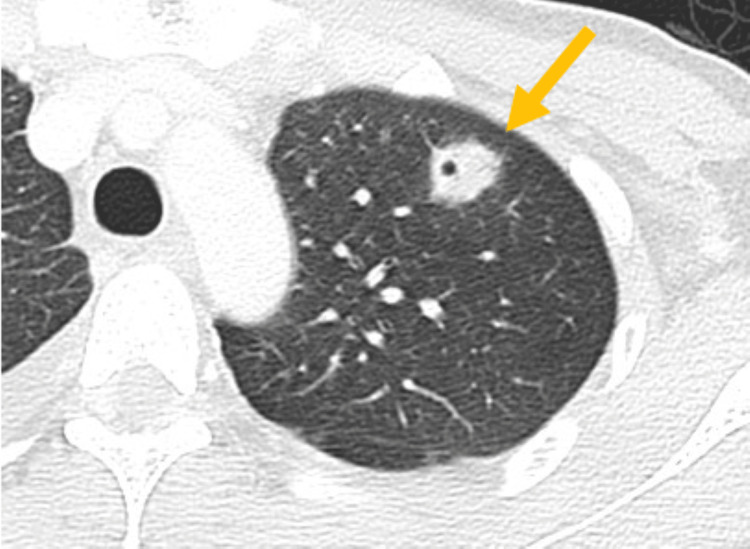
Axial contrast-enhanced chest CT image Axial contrast-enhanced chest CT image obtained on hospital day four demonstrating a peripheral cavitary pulmonary nodule in the left upper lobe (yellow arrow), suggestive of septic pulmonary embolism CT: computed tomography

Intravenous ABPC/SBT was continued, leading to defervescence and a gradual improvement in inflammatory markers (Figure 3). Renal function also improved during hospitalization. After normalization of renal function and hemodynamic stabilization, antimicrobial therapy was transitioned to oral amoxicillin/clavulanate on hospital day 14, and the patient was discharged on hospital day 17. Follow-up CT at the outpatient visit demonstrated complete resolution of the pulmonary nodule (Figure 4). Antimicrobial therapy was discontinued after a total treatment duration of four weeks.

**Figure 3:**
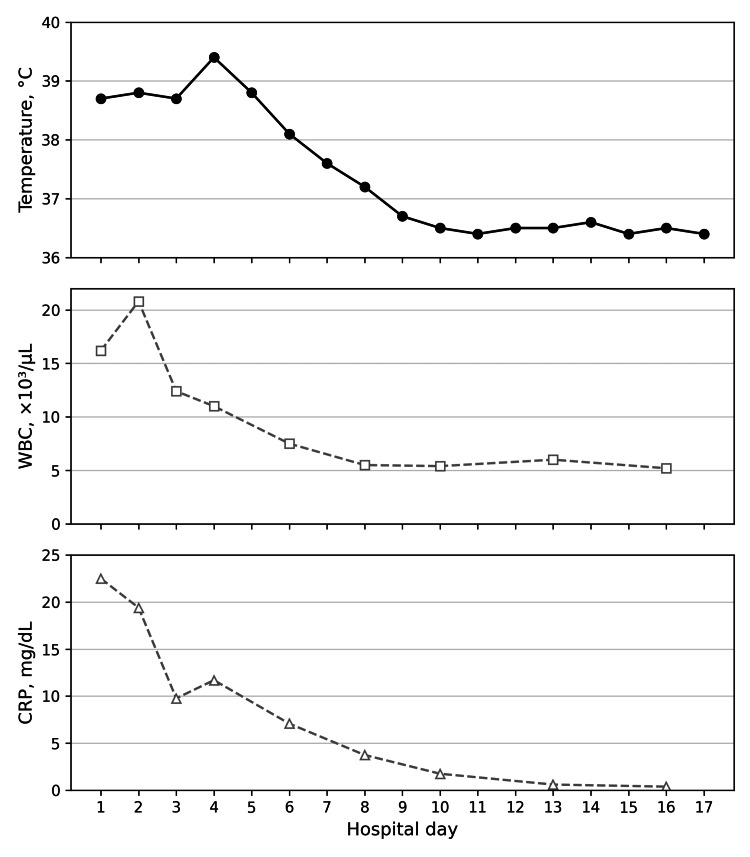
Clinical course of the patient The upper panel shows body temperature, the middle panel shows white blood cell (WBC) count, and the lower panel shows C-reactive protein (CRP) levels during hospitalization. All parameters gradually improved following antimicrobial therapy

**Figure 4:**
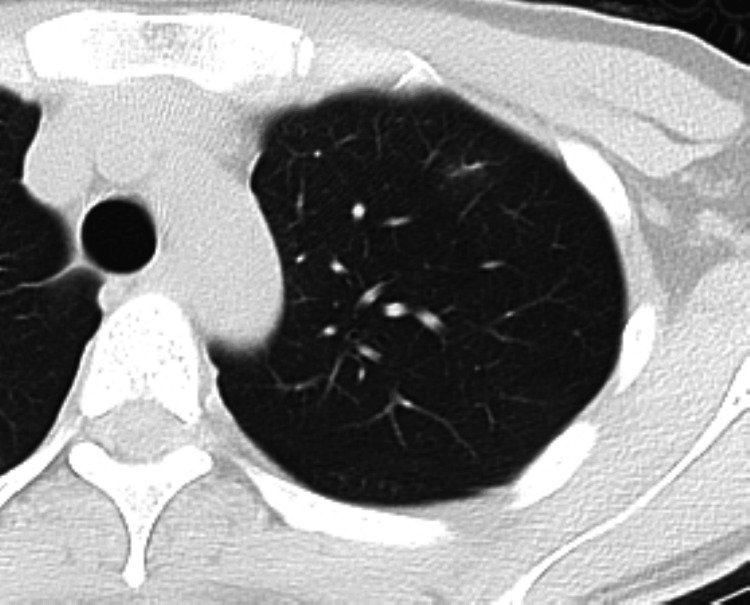
Axial chest CT image at follow-up The previously identified cavitary pulmonary nodule in the left upper lobe is no longer visible, indicating complete radiological resolution CT: computed tomography

## Discussion

The present case demonstrated several clinically important features of Lemierre’s syndrome, including a biphasic clinical course following a transient improvement in pharyngotonsillitis and *F. necrophorum* bacteremia with septic pulmonary emboli. Despite the marked reduction in mortality achieved with antimicrobial therapy, Lemierre’s syndrome remains associated with reported mortality rates of approximately 2-9% [1,2,6,9]. Furthermore, apart from younger age, few risk factors have been consistently identified. Recurrent tonsillitis leading to tonsillar scarring, Epstein-Barr virus infection, and other host or microbial factors have been proposed as potential predisposing conditions; however, their exact roles remain unclear [1,4].

Although the classic form of Lemierre’s syndrome is characterized by antecedent oropharyngeal infection with IJV thrombosis, accumulating reports suggest that the clinical spectrum may be broader than originally recognized. Reported atypical presentations include abdominal variants associated with portal vein thrombosis, variants involving thrombosis at uncommon venous sites such as the external jugular vein or cavernous sinus, cases with disseminated abscess formation, cases caused by non-classical pathogens such as *Klebsiella pneumoniae* and MRSA, and cases arising from infectious foci outside the oropharynx [10-13,15-20].

These observations suggest that the thrombotic manifestations of Lemierre’s syndrome are not necessarily confined to the IJV. Furthermore, IJV thrombosis may be easily overlooked, and even when present, it may not persist throughout the clinical course [8]. As a result, reliance on IJV thrombophlebitis as a mandatory diagnostic criterion may exclude a substantial proportion of otherwise typical cases [1]. Accordingly, there is growing consensus that demonstration of IJV thrombosis is not required for the diagnosis of Lemierre’s syndrome [1,2,7,8]. Our patient had preceding pharyngotonsillitis, blood cultures positive for *F. necrophorum*, and radiologically confirmed septic pulmonary emboli. Although no IJV thrombosis was identified on contrast-enhanced CT or ultrasonography, the overall clinical and microbiological findings were consistent with Lemierre’s syndrome.

In most cases, the initial manifestations of Lemierre's syndrome consist of fever and sore throat, which are clinically indistinguishable from pharyngotonsillitis caused by other viral or bacterial pathogens [1,21]. After this seemingly uncomplicated phase, the second stage, characterized by septicemia and metastatic complications, typically develops several days after the initial oropharyngeal infection [1,2,4]. The proposed mechanism is that antecedent viral or bacterial pharyngitis disrupts the pharyngeal mucosa and lateral pharyngeal space, thereby facilitating invasion by the causative organism and progression to the second stage of infection [3-5].

This biphasic progression may be explained by disruption of the pharyngeal mucosal barrier and extension of infection into the lateral pharyngeal space, facilitating invasion by the causative organism and progression to septicemia and metastatic complications. In our case, preceding streptococcal infection may have impaired the mucosal barrier, permitting secondary invasion by *F. necrophorum*. By the time sepsis becomes clinically apparent, the preceding pharyngeal symptoms have often begun to subside or may have completely resolved, potentially contributing to delayed diagnosis [1,3]. In this case, although the RADT for group A *Streptococcus* was positive, concomitant infection or colonization cannot be excluded. The initial presentation may therefore have represented a polymicrobial process involving both group A *Streptococcus* and *F. necrophorum*. Furthermore, *F. necrophorum* itself is recognized as a potential cause of pharyngitis in adolescents and young adults [22]. This may further complicate the interpretation of a positive RADT result.

In the management of acute pharyngotonsillitis, clinical prediction tools such as the Centor criteria are commonly used to determine the need for RADT for group A *Streptococcus* and for the initiation of antimicrobial therapy [23,24]. However, clear guidance is lacking regarding the management of patients with negative RADT results, and the need for improvement in this area has been highlighted [25]. Centor et al. reported that *F. necrophorum*-positive pharyngitis occurred more frequently than group A β-hemolytic streptococcal pharyngitis among students aged 15-30 years with acute sore throat [26]. Furthermore, Jensen et al. emphasized the significant involvement of *F. necrophorum* in pharyngotonsillitis, particularly in recurrent cases, and recommended anaerobic culture on selective media for throat swabs obtained from patients aged 10-40 years with non-group A streptococcal tonsillitis [27].

Overall, these observations highlight the importance of recognizing *F. necrophorum* as a potential pathogen in acute pharyngotonsillitis, which may, in turn, facilitate earlier recognition of invasive disease. Wright et al. and Pagels et al. have described clinical features, disease course, and laboratory findings suggestive of *F. necrophorum* involvement and progression to Lemierre's syndrome; however, these findings are not necessarily specific to this organism [28,29]. Furthermore, a meta-analysis by Klug et al. demonstrated that even with a cut-off score of 3 or higher on the Centor criteria, the sensitivity for identifying *F. necrophorum* infection was only approximately 30% (Table 2) [26,30-32]. Unfortunately, no validated clinical scoring system for *F. necrophorum* infection is currently available. Clinicians should therefore carefully monitor the clinical course of patients with acute pharyngotonsillitis and remain alert to signs of invasive disease.

**Table 2:** Sensitivity of the Centor criteria for identifying Fusobacterium necrophorum infection This table summarizes the sensitivity of the Centor criteria for identifying Fusobacterium necrophorum infection based on the meta-analysis by Klug et al. [30]

Study				Centor score				
				0	1	2	3	4
Centor et al., 2015 [26]				9%	26%	36%	13%	15%
Hedin et al., 2015 [31]				0%	7%	57%	21%	14%
Kjærulff et al., 2015 [32]				-	50%	17%	33%	0%
In total				5%	26%	38%	18%	12%

An important clinical feature of this case is the biphasic clinical course, in which apparent symptomatic improvement after short-course oral cephalosporin therapy was followed by rapid progression to septic shock and septic emboli. Such transient clinical responses may delay recognition of invasive *F. necrophorum* infection and represent a potential diagnostic pitfall. Previous studies suggest that this type of progression after antecedent antimicrobial treatment is relatively uncommon [2,6]. In Japan, a 10-day course of amoxicillin is recommended as the first-line therapy for group A streptococcal pharyngotonsillitis, whereas short-course oral cephalosporins such as CDTR-PI are sometimes used as alternative regimens in outpatient practice because of their convenient dosing and relatively short treatment courses.

In the present case, the *F. necrophorum* isolated from blood cultures was susceptible to third-generation cephalosporins. However, in Lemierre's syndrome, achieving effective bactericidal concentrations within abscess cavities or septic emboli is often challenging; therefore, antimicrobial therapy typically requires a duration of three to six weeks [1,2,5,7]. As antimicrobial treatment was initially limited to five days, relapse may have occurred after transient suppression of bacterial activity rather than complete eradication. Indeed, after completion of a four-week course of susceptible antimicrobial therapy, the patient's condition improved without recurrence.

As an additional consideration, the role of anticoagulation in Lemierre's syndrome remains controversial [1,3,4,7,8,33]. No randomized clinical trials have been conducted to evaluate the benefits and risks of anticoagulation in Lemierre's syndrome [5,21]. In our case, no IJV thrombosis was identified, and the patient showed clinical improvement with antimicrobial therapy alone; therefore, anticoagulation was deemed unnecessary. Finally, the biphasic clinical course in this patient, characterized by initial symptomatic improvement followed by rapid systemic deterioration, may provide an important clinical clue to the early progression of Lemierre's syndrome. Clinicians should therefore maintain a high index of suspicion for Lemierre's syndrome in patients who develop recurrent fever or systemic deterioration after apparent recovery from acute pharyngotonsillitis, even after appropriate antimicrobial therapy.

## Conclusions

Clinicians should remain vigilant for the potential development of Lemierre's syndrome even when patients with presumed streptococcal pharyngotonsillitis initially improve with antibiotic therapy. A biphasic clinical course, characterized by recurrence or worsening of symptoms after apparent recovery, should serve as a warning sign and should prompt evaluation for deep-seated complications. This report highlights that short-course oral cephalosporin therapy for presumed group A streptococcal pharyngitis does not necessarily prevent the development of invasive *F. necrophorum* infection and may occasionally obscure the early evolution of Lemierre's syndrome.
